# Supplementary material for: Origins of chemoreceptor curvature sorting in Escherichia coli
Source: Nat Commun. 2017 Mar 21;8:14838. doi: 10.1038/ncomms14838 (PMC5364426; doi:10.1038/ncomms14838)
Supplement: Supplementary Information — Supplementary Figures [file ncomms14838-s1.pdf]

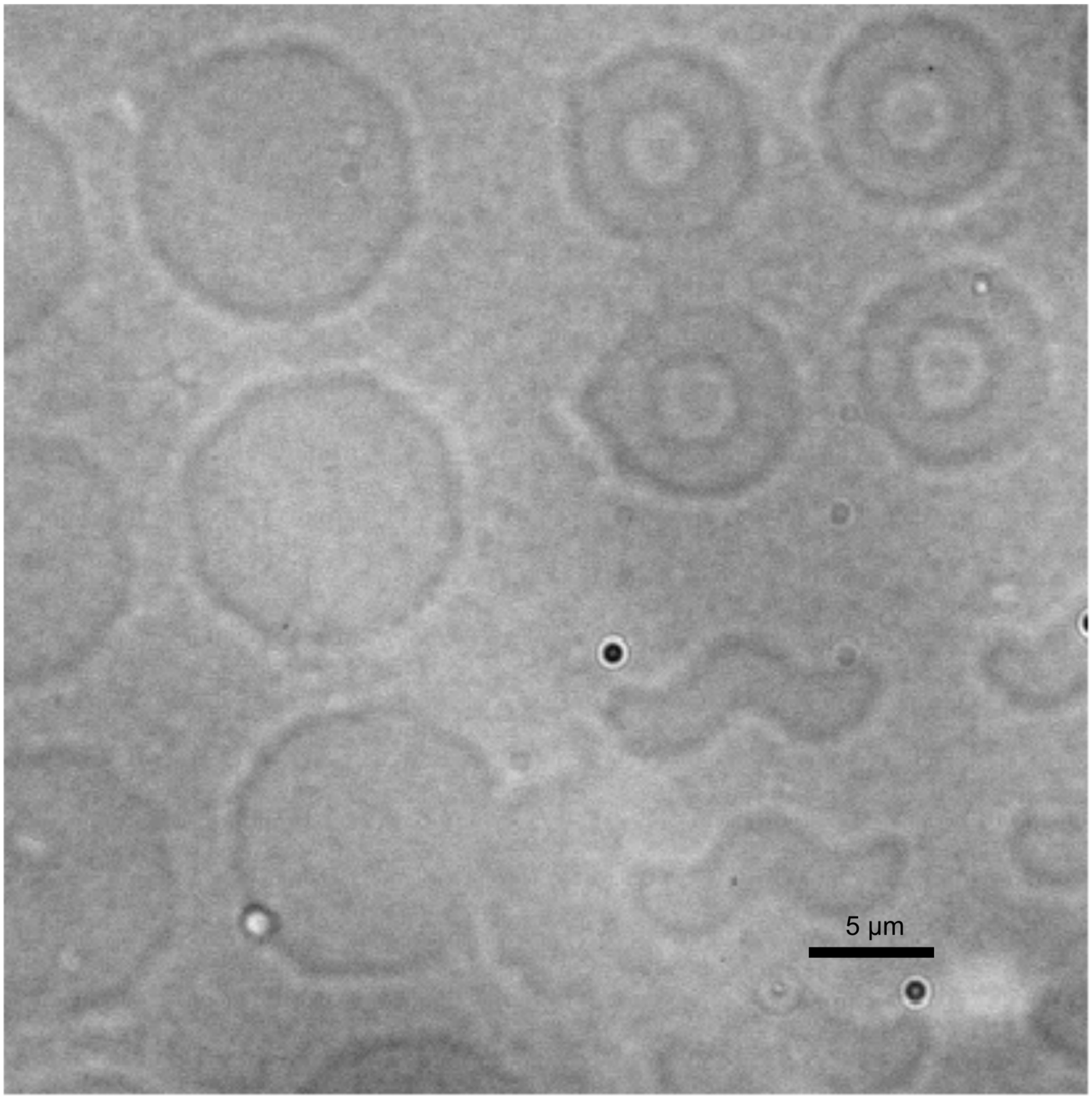


**Supplementary Fig. 1**. Sample agar microchambers.

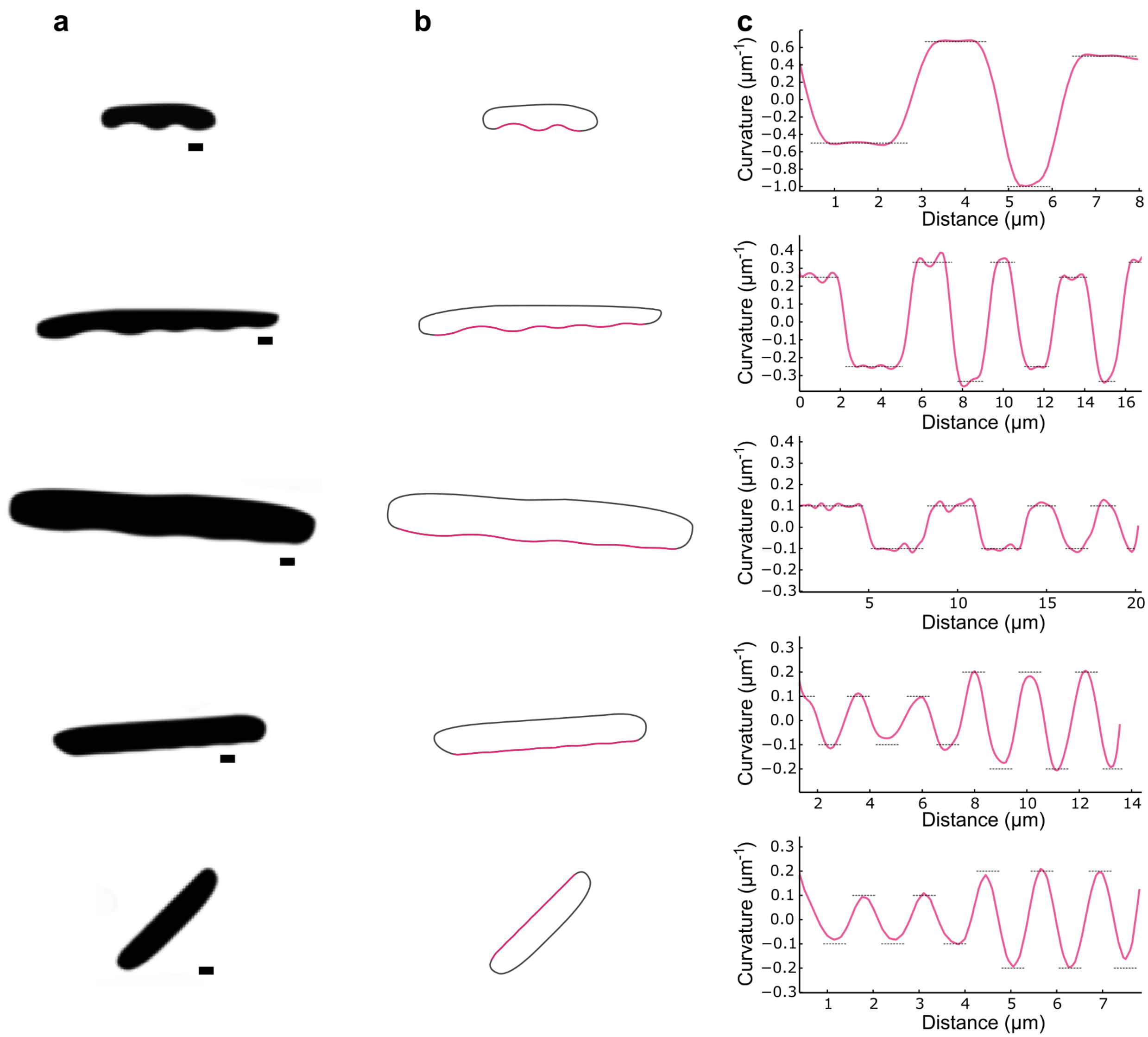


**Supplementary Fig. 2**. Simulated test dataset to validate curvature algorithm. Circles with various radii of curvature were drawn in a vector graphics program, and merged together to make cell like shapes with varying curvature. The shapes were smoothed with a Gaussian filter to simulate a nominal 100x objective resolution limit (250 nm), and then down-sampled to mimic the pixel size of a camera (100 nm). The simulated data, shown in (**a**), was fed through our contour finding algorithm (**b**), and curvature was measured in the resultant traces, shown in (**c**). Dotted horizontal lines in (c) represent the actual curvature of the simulated data. Scale bars represent 1 μm.

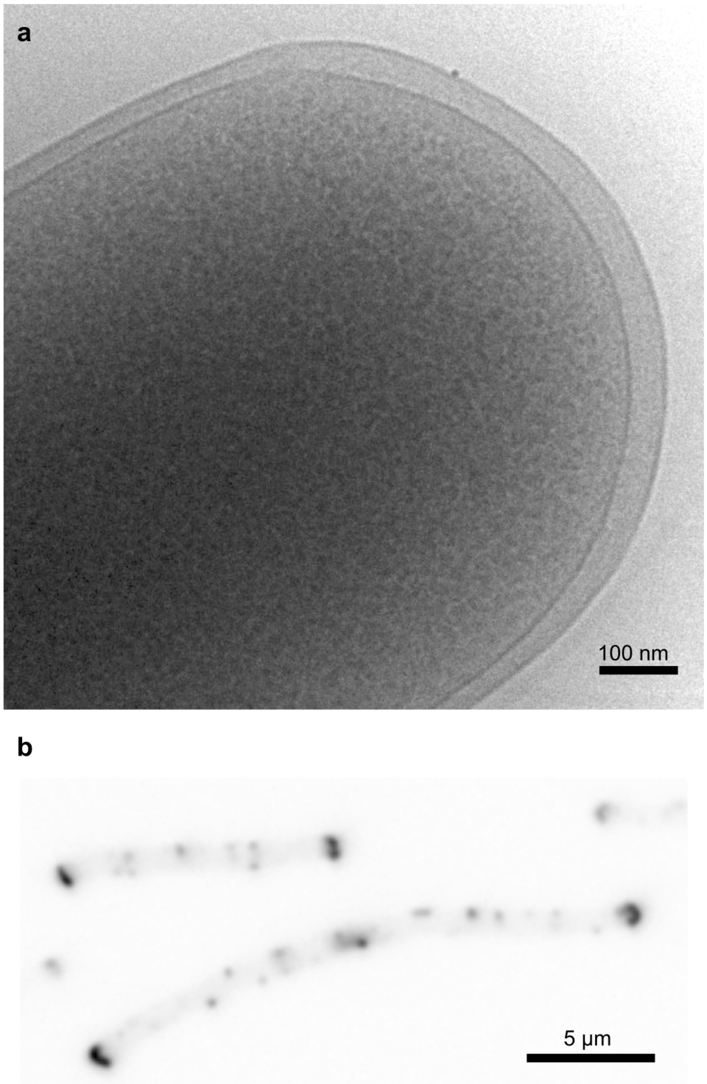


**Supplementary Fig. 3**. Cryo-transmission electron imaging of artificial curvature sensitive complexes. (**a**) 2-D Cryo-transmission electron image of *E. coli* overexpressing synthetic protein clusters composed of TsrΔT-SH3 and 8xSH3. (**b**) Before the cells were flash frozen, protein complexes were imaged using YFP-CheR to confirm overexpression of the receptor scaffold complex.

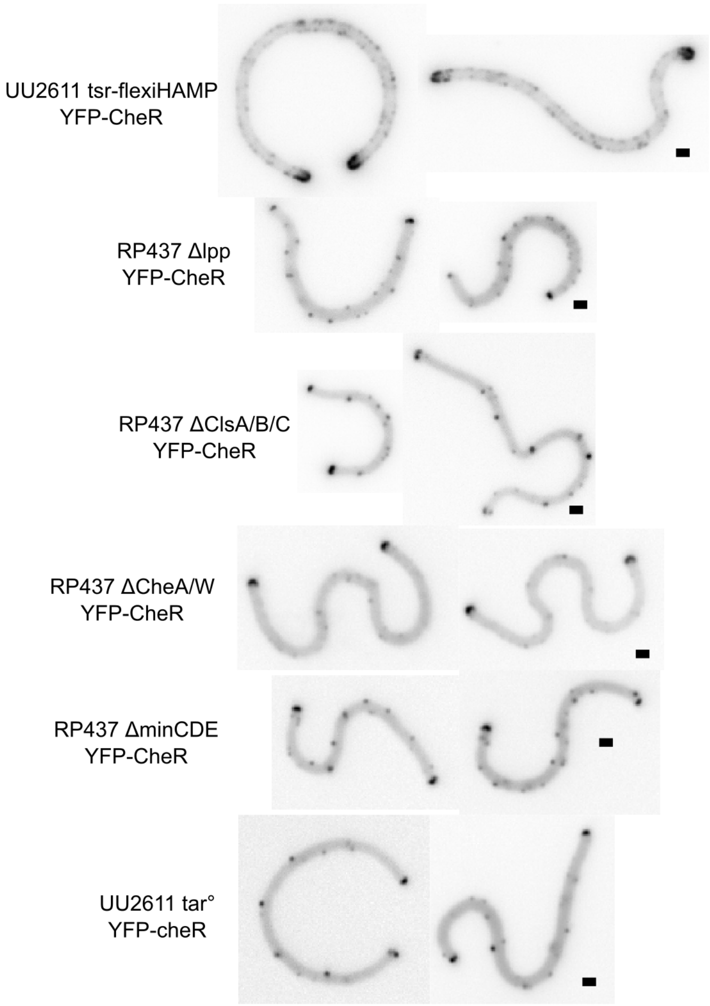


**Supplementary Fig. 4**. Artificially deformed *RP437* mutants. All scale bars 1 μm.

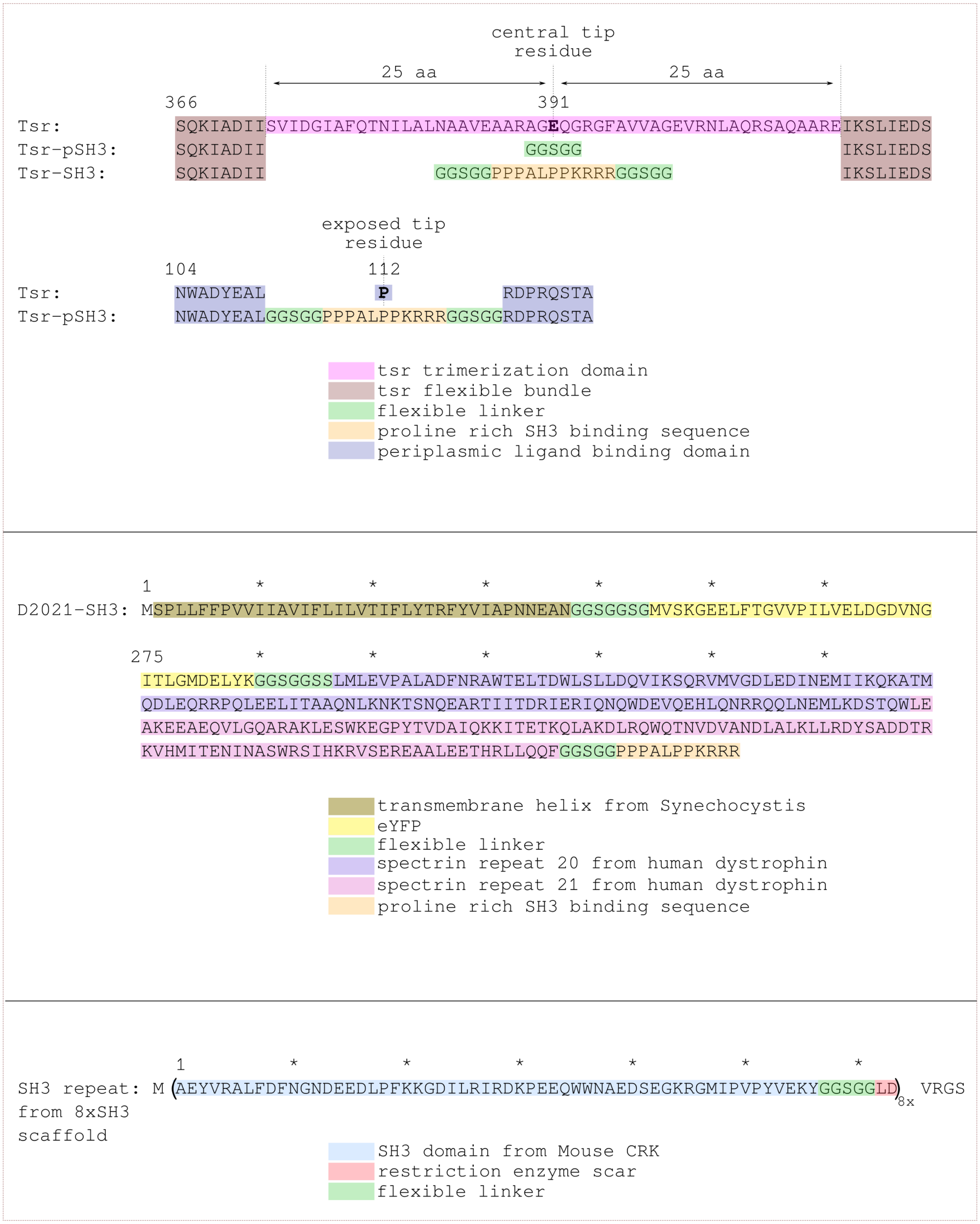


**Supplementary Fig 5.** Synthetic curvature sensitive protein complex protein sequences. Protein sequences of Tsr-SH3 and Tsr-pSH3 are shown aligned against wild-type Tsr, to demonstrate how the insertion/deletions were made.
